# An Update on Prevalence, Assessment, and Risk Factors for Sleep Disturbances in Patients with Advanced Cancer—Implications for Health Care Providers and Clinical Research

**DOI:** 10.3390/cancers14163933

**Published:** 2022-08-15

**Authors:** Gunnhild Jakobsen, Kari Hanne Gjeilo, Marianne Jensen Hjermstad, Pål Klepstad

**Affiliations:** 1 Department of Public Health and Nursing, Faculty of Medicine and Health Sciences, Norwegian University of Science and Technology (NTNU), 7491 Trondheim, Norway; 2 Cancer Clinic, St. Olavs Hospital, Trondheim University Hospital, 7006 Trondheim, Norway; 3 Clinic of Cardiology and Department of Cardiothoracic Surgery, St. Olavs Hospital, Trondheim University Hospital, 7006 Trondheim, Norway; 4 Regional Advisory Unit in Palliative Care, Department of Oncology, Oslo University Hospital, 0424 Oslo, Norway; 5 European Palliative Care Research Centre, Department of Oncology, Oslo University Hospital, 0424 Oslo, Norway; 6 Institute of Clinical Medicine, University of Oslo, 0318 Oslo, Norway; 7 Department of Anaesthesiology and Intensive Care Medicine, St. Olavs Hospital, Trondheim University Hospital, 7006 Trondheim, Norway; 8 Department of Circulation and Medical Imaging, Faculty of Medicine and Health Sciences, Norwegian University of Science and Technology (NTNU), 7491 Trondheim, Norway

**Keywords:** sleep, sleep disturbances, insomnia, advanced cancer, palliative, palliative care

## Abstract

**Simple Summary:**

This review focuses on sleep in patients with advanced cancer. Cancer patients experience multiple symptoms and they receive concomitant medications. These are all factors that may affect sleep. In this paper, we present recommendations on sleep assessment in patients with advanced cancer and highlight cancer-related factors that may contribute to insomnia. Sleep is an essential aspect of health-related quality of life; therefore, it is important for health care providers to focus on sleep to improve patient care.

**Abstract:**

Patients with advanced cancer experience multiple symptoms, with fluctuating intensity and severity during the disease. They use several medications, including opioids, which may affect sleep. Sleep disturbance is common in cancer patients, decreases the tolerability of other symptoms, and impairs quality of life. Despite its high prevalence and negative impact, poor sleep quality often remains unrecognized and undertreated. Given that sleep is an essential aspect of health-related quality of life, it is important to extend both the knowledge base and awareness among health care providers in this field to improve patient care. In this narrative review, we provide recommendations on sleep assessment in patients with advanced cancer and highlight cancer-related factors that contribute to insomnia. We also present direct implications for health care providers working in palliative care and for future research.

## 1. Introduction

Despite advances in treatment, cancer continues to cause substantial morbidity and mortality. For patients with a life-threatening disease, issues regarding quality of life for their remaining lifetime are critical [1]. Patients with advanced cancer experience multiple symptoms of fluctuating intensity and severity during the disease trajectory [2,3]. They normally use multiple concomitant medications, including opioids, which together with adverse symptoms may affect sleep [4,5,6,7]. Sleep is an essential aspect of health-related quality of life, and thus it is important to gain knowledge in this field to improve patient care. In this narrative review, we present current knowledge that relates to sleep disturbances and sleep assessment in patients with advanced cancer, defined as cancer that is unlikely to be cured and that may have spread from the original site to other parts of the body [8]. In addition, we highlight cancer-related and other factors which may contribute to insomnia in these patients. We focus on the implications for health care providers working in palliative as well as recommendations for future clinical research.

## 2. Sleep Disorders and Sleep Disturbances

According to the International Classification of Sleep Disorders, third edition (ICSD-3) of the American Academy of Sleep Medicine, sleep disorders are grouped into six major categories: insomnia, sleep-related breathing disorders, central disorders of hypersomnolence, circadian rhythm sleep–wake disorders, parasomnias, and sleep-related movement disorders [9]. In this paper, we use the term “insomnia in the context of cancer”, as proposed by Savard and Morin: [10]

(1) Difficulty initiating sleep (greater than 30 min to sleep onset) and/or difficulty maintaining sleep (greater than 30 min nocturnal waking time);
(2) Sleep difficulty at least 3 nights per week;
(3) Sleep difficulty that causes significant impairment of daytime functioning.

Although there is a clear statement of insomnia as a sleep disorder, several non-specific terms are used for sleep by researchers, clinicians, and the public. The term «sleep disturbances» is used to designate insufficient or excessive sleep duration or poor self-reported sleep quality and may refer to sleep related symptoms and signs regardless of whether they fulfil criteria for specific diagnoses or not [11,12]. Another term which also lacks definitional consensus is «sleep quality». Consequently, sleep continuity measures such as sleep latency, awakenings, wake after sleep onset, and sleep efficiency are used as indicators of sleep quality [13]. For instance, shorter sleep latencies, fewer awakenings, and reduced wake after sleep onset indicate good sleep quality. The patient’s subjective experience of sleep quality, as for instance reported on a numerical rating scale, can also be considered to describe sleep quality. Poor sleep quality is a subjective phenomenon and may be described by individual patients as a disruption of their habitual sleep pattern, difficulty falling asleep, frequent awakening, or nonrestorative sleep [14]. This review embraces both aspects of sleep quality; the patient-reported overall global approach of each night’s sleep and quantitative aspects of sleep, such as total sleep time and sleep onset latency (i.e., how many minutes it takes to fall asleep starting from the moment of intention to fall asleep). However, health care providers should be aware of other sleep-related issues as the ones mentioned above, such as excessive daytime sleepiness, circadian rhythm disorders, or sleep-disordered breathing in cancer patients.

## 3. Sleep Assessment

To obtain detailed information on sleep disturbances, it is recommended to examine sleep by combining subjective methods using patient-reported outcome measures (PROMs) and objective registrations such as polysomnography (PSG) and actigraphy [15,16,17,18].

PROMs of sleep include sleep diaries and questionnaires [19]. A structured sleep diary is used by patients to register their bedtime hour, time to fall asleep, number and duration of awakenings during the night, and time of morning awakening and arising from bed [15,20]. In an expert consensus statement Carney et al. concluded that standardized, patient-informed sleep diaries are the standard for subjective sleep assessments [20]. In routine clinical care, a questionnaire such as the revised Edmonton Symptom Assessment System (ESAS-r) is recommended to screen for sleep disturbances in patients with advanced cancer [21]. ESAS-r is a valid and reliable questionnaire for the assessment of the intensity of symptoms in cancer populations, where the severity of each symptom is rated from 0 to 10 on a numerical scale, with 0 meaning that symptom is absent and 10 meaning that it is of the worst possible severity [22]. The ESAS-r consists of nine core symptoms (pain, tiredness, nausea, depression, anxiety, drowsiness, appetite, feeling of well-being, shortness of breath, and an optional 10th symptom to be selected by patients). Today, sleep is not a part of the ESAS-r symptoms, and the optional 10th symptom is often used to assess sleep. For screening purposes, Yennurajalingam et al. suggests that a cut-off of greater than or equal to four should generate further assessment of sleep [21,23].

Another questionnaire, which is validated and widely used to assess sleep quality in patients with advanced cancer is the Pittsburgh Sleep Quality Index (PSQI) [15,24]. It includes seven components of sleep: sleep quality, sleep latency, sleep duration, sleep efficiency, sleep disturbances, use of sleep medications, and daytime dysfunction. The component scores are summed to obtain a global sleep score ranging from 0 to 21, with higher scores indicating worse sleep quality [24]. Using this tool might improve the understanding of sleep difficulties experienced by cancer patients [25]. It covers multiple aspects relevant to sleep quality and might clarify the effect of sleep disturbances on patients’ daily life. In addition, it is simple to use in clinical practice with a completion time of 5 to 10 min [26]. Other examples are the Insomnia Severity Index (ISI) and Athens Insomnia Scale (AIS). The ISI measures patients’ perception of insomnia [27]. It is composed of seven items that evaluate the severity of sleep-onset, sleep maintenance, early morning awakening, satisfaction with current sleep pattern, interference with daily functioning, noticeability of impairment attributed to sleep problems, and level of distress caused by the sleep problems. The AIS is a self-assessment instrument, designed for quantifying sleep difficulty based on the ICD-10 criteria [28]. Health care providers in palliative care can use either ISI or AIS for quick identification of potential sleep problems in an individual cancer patient [29]. Thus, the use of such questionnaires in routine clinical care may help health care providers to gain insights into the patients’ sleep problems.

The specific PROMs for the assessment of sleep vary in relation to which period they are designed to cover. For instance, the ESAS-r is typically used for the assessment of sleep last night [21], while the PSQI is designed to assess sleep last month [24]. The time interval in the ISI is the last two weeks [27], and the AIS is during the last month, or some other period of time, whose length depends on the purpose of a given study [28]. Thus, different studies with different aims and time frames may use different PROMs for sleep assessments.

Today, PSG is the gold standard for measuring sleep [15,30]. However, PSG is a comprehensive assessment method that provides overnight measures of brain waves, eye movement, muscle tension, electrocardiogram, and respiratory parameters. The PSG instrument is a complex monitoring device which requires specially trained personnel to attach the patients to its multiple sensors. As such, this method is usually too demanding for patients with a hight symptom burden, even in a study setting, let alone in routine care [31]. However, several studies have used actigraphy in the monitoring of sleep in patients with advanced cancer [32,33,34]. An actigraph, also known as an actometer, is worn on the wrist or ankle to record acceleration or deceleration of body movements, which indirectly indicates the state of sleep or wakefulness [35,36]. Advantages of actigraphy over PSG include ease of use, inexpensive recordings over extended periods of days, weeks, or months, and usefulness in cognitive impaired patients where PSG is not possible [37]. For seriously ill patients, such as patients with advanced cancer, actigraphy has become a valuable tool for objective sleep assessment [17].

Actigraphy is also a validated method to evaluate circadian rhythms both in research and clinical settings [16,38]. A recent review analysed the rest-activity circadian rhythm disruption in advanced cancer patients [39]. Circadian disruption was reported to be prevalent in this patient population. The disruption was manifested as lower activity levels during the day, more frequent and longer daytime naps, and fragmented night-time sleep. The circadian process is an internal rhythm or clock that dictates periods of activity (wakefulness) and inactivity (sleep) based in a light–dark cycle, and sleep is one of many bodily functions under control of the circadian clock [40,41]. As altered patterns have been described for several circadian rhythms in cancer [42], it is important to evaluate circadian rhythms in these patients. In fact, a study among patients with advanced cancer reported statistically significant and clinically meaningful associations between circadian rest–activity rhythm alterations and the severity of fatigue and anorexia, as well as impairment of physical and social dimensions of health-related quality of life [43]. This supports the need to develop interventions that target the circadian clock to improve symptom control in these patients.

## 4. Prevalence of Poor Sleep Quality

Sleep disturbances are prevalent in cancer [44,45,46,47]. A recent meta-analysis on the prevalence of sleep disturbances in patients with cancer reported an overall prevalence of 60.7%, suggesting that more than half of the cancer patients experience sleep disturbances [44]. Most importantly, the prevalence was even higher in patients with advanced cancer, with an overall prevalence of 70.8% [44]. Insomnia is considered an underdiagnosed and undertreated health problem in palliative care [48], as about one third of patients with cancer has insomnia symptoms. This is about three times higher than in the general population [45,49,50].

At the same time, the prevalence of patient-reported sleep disturbances in advanced cancer differs largely across studies. Table 1 provides examples of studies that have examined patient-reported sleep prevalence rates in patients with advanced cancer and the different assessment tools being used [21,25,51,52,53,54,55,56,57,58,59]. Such differences may be due to different study methods, designs and aims, assessment tools used, and population characteristics. In addition, and as mentioned above, the term «sleep disturbances» is non-specific and may contribute to the different prevalence rates across studies.

Jakobsen et al. demonstrated that the majority (78%) of 604 adult patients with cancer pain using WHO Step III opioids reported poor sleep quality using the PSQI [51]. All components of sleep quality were affected suggesting that patients with advanced cancer experience a mixture of sleep disturbances, including difficulty initiating sleep, staying asleep, early awakenings, and that external factors such as pain, having to use the bathroom, inability to breath comfortably, or feeling too cold or hot disturbed sleep [51]. In line with other studies in palliative care [52,53,58,60], the mean PSQI global score was 8.8 (±4.2; range 0–20). Overall, studies demonstrate that sleep disturbances in patients with advanced cancer are prevalent and represent a complex clinical situation in palliative care.

## 5. Predisposing Factors for Insomnia in Advanced Cancer and Consequences for Other Symptoms

**Figure 1:**
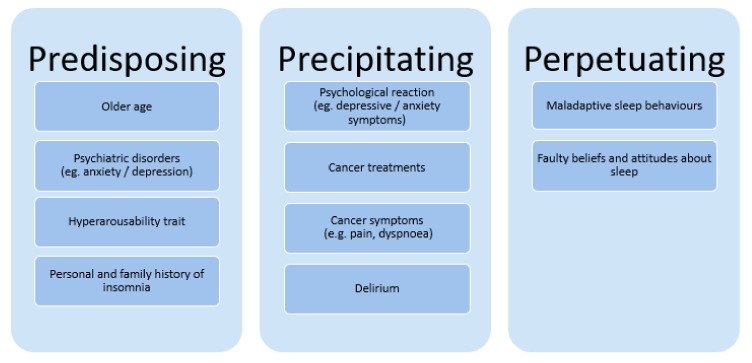
Insomnia in the context of advanced cancer. Examples of predisposing, precipitating, and perpetuating factors involved in the development of insomnia in advanced cancer [10,46,48,62,63].

The potential causes of sleep disturbances in patients with advanced cancer are many, varied, and complex [10,46,61]. Clearly, the cancer disease and cancer treatment, place patients at increased risk for disruption of normal behaviors, habits, and physiological states that normally lead to restful sleep. For insomnia, several etiologic factors are involved in patients with advanced cancer. These are grouped into three main categories: predisposing factors, precipitating factors, and perpetuating factors [10,46,48,62,63]. Figure 1 illustrates some of these factors.

Predisposing factors increase the individual’s general vulnerability to develop insomnia, among these are older age, hyperarousability as trait, and personal or familiar history of insomnia [10,62,63]. Patients who have had insomnia prior to their cancer are at increased risk of experiencing insomnia when they are faced with cancer [62]. On the other hand, contrary to the general population, where female gender is a known predictor of insomnia, gender does not seem to be a predictor if insomnia in patients with advanced cancer [48].

Precipitating factors or situational conditions trigger the onset of insomnia, in which cancer is characterized by a succession of severe stressors that can trigger insomnia at any time during the cancer trajectory [48,64,65]. Precipitating factors include cancer treatments that can alter levels of inflammatory cytokines, disrupt circadian rhythms or sleep–wake cycles or cause menopause. Moreover, hospitalization, in itself, disturbs sleep [66]. Finally, medications used to treat or manage side effects and cancer-related symptoms, such as opioids or corticosteroids, will influence sleep [10,46,64]. However, in patients with advanced cancer, it may be difficult to differentiate, for instance, corticosteroid adverse effects from symptoms related to a progressive malignant disease [57,67]. To illustrate, treatment with methylprednisolone 16 mg twice daily for 7 days in patients with advanced cancer did not result in more patient-reported sleep problems as measured by the European Organisation for Research and Treatment of Cancer Quality of Life Questionnaire Core 30 in a randomized, placebo-controlled, double-blind trial using a standardized dose of corticosteroids [68].

In recent years, there has been an increased interest among researchers to understand the association between sleep problems and cancer-related symptoms. These symptoms are also referred to as precipitating factors for insomnia [10,62,69,70]. Patients with advanced cancer often report high levels of several co-occurring symptoms, in which pain, fatigue, nausea and vomiting, dyspnoea, constipation, loss of appetite, and depression are among the most common symptoms [71,72]. Pain is one of the most frequently reported cancer-related symptoms in association with sleep disturbances in patients with advanced cancer. In line with other studies in palliative care [5,21,56,58], an international multi-centre study reported that more pain was significantly associated with poor sleep as measured by the PSQI, and that pain intensity was a statistically significant predictor of poor sleep in patients with advanced cancer [51].

Another cancer-related symptom, psychological distress, is associated with sleep disturbances in patients with advanced cancer. Sleep quality, as assessed by the PSQI, was associated with emotional functioning in patients with advanced cancer using WHO Step III opioids [51], suggesting that patients with lower scores in emotional function, i.e., feeling tense, being worried, being irritable, and feeling depressed, reported more sleep disturbances. These results tie well with previous studies in palliative care which have demonstrated that sleep disturbances are associated with depression and reduced quality of life [58,73,74,75,76]. To illustrate, excessive rumination, potentially involved in increased psychological distress in palliative care, was associated with insomnia in patients with advanced cancer [74]. In addition, sleep disturbance has been suggested as a mediator of the relationship between respiratory symptoms and quality of life in patients with advanced lung cancer [77]. Overall, these findings suggest that emotional function and sleep may be related. However, it is important to recognize that these studies all report associations. A causal effect is therefore not established. For instance, pain may induce disturbed sleep, disturbed sleep may increase the experience of pain, or a shared factor may cause both disturbed sleep and increased pain.

Perpetuating factors include behavioral factors such as excessive daytime sleeping, and maladaptive cognitions, i.e., inaccurate appraisal of sleep [46]. Patients with insomnia might have several faulty beliefs and attitudes about sleep and sleepiness that may contribute to maintaining the sleep problem over time [10]. For cancer patients, this might lead to thoughts such as “If I don’t sleep well, my cancer will come back” [10]. Savard and Morin suggested that maladaptive sleep habits, which develop in response to sleep disturbances, are the most salient factors in the maintenance of insomnia [10]. These factors are responsible for increasing physiological, cognitive, and emotional arousal and performance (the pressure to sleep) [62]. To overcome cancer-related fatigue, patients are often advised by health care providers to rest during the day. A single short afternoon nap may not have negative impact on night-time sleep. However, extensive daytime napping and increased time spent in bed might result in irregular sleep–wake patterns. Furthermore, the long-term consequences involve desynchronization of the sleep–wake cycle [10,62]. Thus, excessive daytime sleeping may contribute to the maintenance of insomnia.

## 6. Implications for Clinical Practice and Future Research

Overall, this review of sleep quality in patients with advanced cancer highlights the importance of sleep in several relevant areas for health care providers and researchers working in oncology and palliative care.

### 6.1. Prevalence of Poor Sleep Quality

Knowledge of the large proportion of patients experiencing poor sleep quality in advanced cancer is important for health care providers. This cohort represents a large number of patients all over the word. Thus, the high prevalence of poor sleep and the mixture of sleep disturbances in these patients call for awareness of sleep quality in daily routine care. Given the high level of physical and psychological symptoms in these patients, health care providers should be aware of the prognostic consequences of sleep disturbances. A recent review indicated that disturbed sleep during oncological treatment might be a relevant behavioral marker of poor cancer prognosis [78]. In detail, disturbances in sleep and sleep–wake activity immediately prior to or during treatment were associated with reduced overall survival, poorer response to treatment, and shorter time to progression [78]. Moreover, sleep disorder prevalence data might be helpful for future development of interventions in the treatment of sleep disturbances. Thus, in palliative care research, sleep prevalence data should be elicited from large samples of patients with advanced cancer to reflect the multitude of cancer-related factors that might affect sleep quality. Clinical studies should incorporate sleep questionnaires to advance the knowledge in this field and to improve care.

### 6.2. Cancer-Related Factors for Insomnia in Advanced Cancer

This review highlights important cancer-related factors that contribute to poor sleep quality in patients with advanced cancer. Despite previous findings of a relationship between sleep and cancer-related symptoms [53,54,55,60,69], it is difficult to predict which patients will develop sleep disturbances in palliative care. Individual factors, including both cancer-related and other symptoms as well as psychological factors, interfere with how the patient handles the cancer disease and how they manage their sleep. This also influences to what extent they report sleep disturbances as a problem and how much attention and treatment they want. Taken together, all of this has an impact on sleep quality. However, health care providers may use this knowledge to identify vulnerable patients with an increased risk of sleep problems. Moreover, findings on the associated and predictive factors of sleep quality are of importance when developing appropriate management and/or preventive strategies. Knowledge of how sleep affects daytime functioning is important in patients with life-threatening illness, as restorative sleep is necessary for healing, recovery, and to fight and resist infections [79].

To improve the scientific knowledge of sleep, and to identify risk factors for poor sleep quality in advanced cancer patients, it is useful to examine the relationship between sleep and cancer-related factors in palliative care research. However, interpretations of causality are not possible from cross-sectional designs, which uses estimation of association between variables. In cross-sectional studies it is difficult to establish if sleep problems are simply associated with cancer-related symptoms, or whether a sleep problem in itself elicits symptoms and should be the main target to alleviate symptoms like pain, depression, anxiety, and distress. Hence, the impact of poor sleep on daytime functioning is difficult to establish. To illustrate, the relationship between pain and sleep is reported as bidirectional and reciprocal [80]. Therefore, it might be useful to ask whether this represents a vicious circle in patients with advanced cancer, in which poor sleep quality affects daytime functioning, or even daytime symptom intensity, which in turn affects night-time sleep quality.

Thus, future research in palliative care should investigate daytime consequences of poor sleep quality in advanced cancer. Here, symptom clusters are relevant. Insomnia was recently identified, together with pain and emotional functioning, in terminally ill patients with cancer [81]. Another study identified insomnia as part of a neuropsychological cluster together with depression and anxiety [82]. Future studies should investigate if these symptoms could be treated concomitantly. Consequences of poor sleep will be important to establish, using longitudinal design, to avoid the risk that research on sleep in patients with advanced cancer is limited to correlational science in cross-sectional studies and the low level of evidence in clinical decision making from such studies.

### 6.3. Sleep Assessment

The knowledge of the complexity of sleep disturbance in advanced cancer, including difficulty initiating sleep, staying asleep, and early awakening is relevant for the understanding of how to categorize poor sleep to address each patient’s individual sleep disturbances. Thus, to identify and treat patients with sleep disturbances, health care providers working in oncology and palliative care should routinely assess sleep problems [83].

Oncology nurses can play a leading role in addressing sleep problems, as they often spend more time with patients experiencing cancer-related symptoms than any other health professionals. Thus, it is of utmost importance that oncology nurses have knowledge of sleep assessment to provide good symptom control. However, a study on sleep assessment in patients living with cancer, discovered that few nurses assessed sleep patterns, undertook further assessment and investigations for patient’s sleep problems, or reassessed the patients sleep patterns in case the patient complained of non-efficacy of the interventions [84].

Several reasons might explain the lack of sleep assessment among health care providers. Sleep disturbances might be considered a low-priority problem compared to the cancer itself, or because of a lack of sleep assessment protocols or guidelines [84,85]. Interestingly, a literature review on nurses’ perceptions of sleep in the intensive care unit revealed that intensive care unit nurses lack a complete understanding of the importance of sleep [86]. Moreover, Ye et al. identified limited understanding of the importance of sleep during hospitalization, the lack of standardized assessment tools for sleep, lack of education in sleep evaluation, inadequate interdisciplinary communication, and lack of supportive hospital infrastructure as barriers to the effective management of sleep [87]. Knowledge about sleep and its physiology is, in many cases, based on personal experience and common sense rather than being evidence based [88]. Thus, one important aspect in sleep assessment is that health care providers have insufficient knowledge about sleep. Fortunately, the problems are now being addressed, and several studies argue that sleep should be a topic included in nursing education and training [89,90,91].

Although, beyond the scope of this review, we would like to mention that the management of sleep disturbances in patients with advanced cancer lack evidence-based knowledge in palliative care [46,48,92]. Nevertheless, it is important to be aware of this gap in knowledge. The sparse evidence and clear guidelines for treatment of sleep problems in patients with advanced cancer may contribute to health care personnel’s reluctance to address sleep problems. In addition, it may also explain the lack of systematic assessment in the first place. One might question whether screening for sleep disturbances is meaningful when there is limited treatment to offer. Some will even find it unethical to systematically screen for sleep problems given the lack of evidence-based knowledge of pharmacological and non-pharmacological treatment in palliative care. On the other hand, we recommend that sleep problems should be assessed as an inherent part of most other prevalent symptoms in this group of patients.

Patients themselves might also contribute to the underassessment of sleep disturbances. Despite many patients expressing concerns about sleep, these problems are not always discussed with health professions during oncology appointments [93]. Patients do not usually report their sleep problems to health professions because in general it is seen as less significant than the cancer [94]. In addition, there is a perception by some patients that health professionals do not want to hear about it [94]. Some patients even believe that healthcare workers are too busy to treat such an insignificant problem [95]. To examine these thoughts might give valuable insights into how patients cope with sleep disturbances. A qualitative study revealed that patients with chronic heart failure used different self-care strategies to promote sleep [96]. However, these strategies were based on common knowledge, and did not follow any common methods. It is important that health care providers are informed about such self-care strategies. This is useful information in order to guide patients about the benefits of using more evidence-based approaches [97]. Thus, patients with advanced cancer should be asked about sleep. Patients should be encouraged to discuss their sleep problems with members of the healthcare team in palliative care.

Future studies on sleep in patients with advanced cancer care should combine PROMs and objective registrations of sleep. As the use of both actigraphy and PROMs is recommended in patients with advanced cancer, a sole use of PROMs can result in a lack of important information on sleep [30,31,32,98]. In addition, to gain knowledge of patients’ perspective in palliative care research, clinical evaluation of insomnia should incorporate qualitative assessments of issues relevant to the patient’s subjective experience of insomnia [99,100].

### 6.4. Treatment

The treatment plan for sleep disturbances in patients with advanced cancer should address the multifactorial and treatable causes. Thus, a combined stepwise pharmacological and non-pharmacological approach is recommended [92]. Symptom control should be the first step to remove the causative condition if possible (e.g., pain, dyspnoea, and anxiety) [101]. The second step should include non-pharmacological sleep interventions with cognitive and behavioral therapy for insomnia (CBT-I) [46]. This treatment incorporates cognitive and behavior-change techniques and targets dysfunctional attitudes, beliefs, and habits involving sleep [102]. Bright-light therapy is also used to improve sleep, but to our knowledge not formally tested in advanced cancer patients [103]. Short-term pharmacological treatment may be necessary until CBI-I takes effect or for those being refractory to CBT-I. A recent systematic review of the treatment of insomnia in palliative care identified hypnotics, antidepressants, and antihistamines as pharmacological treatment options of sleep disturbances [48]. However, evidence-based knowledge about the best pharmacological treatments for insomnia in patients with advanced cancer are scarce. For practical purposes, the palliative care network of Wisconsin has provided an overview of the pharmacological treatment of insomnia [104]. When pharmacological treatment is used, the choice of the specific agent within a class should be directed by factors such as symptom pattern, treatment goals, past treatment responses, and the presence and significance of contraindications [46].

## 7. Conclusions

The overall aim of this review is to contribute to evidence-based knowledge of sleep in patients with advanced cancer. The high prevalence of poor sleep quality and the mixture of sleep disturbances in these patients calls for awareness in health care providers. To identify and treat patients with sleep disturbances, health care providers should routinely assess sleep problems using PROMs. Patients with advanced cancer should be asked about sleep. More importantly, we should encourage patients to discuss their sleep problems and sleep-related concerns with formal and informal caregivers in hospital as well as at home. Further characterization of sleep disturbances in patients with advanced cancer is needed, with particular emphasis on contributing factors, such as cancer-related symptoms. Thus, more research using robust longitudinal designs with a comprehensive assessment of sleep is necessary. A better understanding of the relationship between cancer-related symptoms and sleep enhances the possibilities of developing more targeted interventions, which will increase the scientific basis for knowledge in the treatment of sleep disturbances in palliative and oncology care.

## Figures and Tables

**Table 1:** Examples of studies investigating sleep quality in patients with advanced cancer [21,25,51,52,53,54,55,56,57,58,59].

Author, Country (Year)	N	Prevalence of Poor Sleep ^1^	Questionnaire
Mercadante, Italy (2021) [59]	182	50%	Athens Insomnia Scale
Jakobsen, Denmark, Germany, Lithuania, Norway, Switzerland (2018) [51]	604	78%	PSQI
Collins, USA (2017) [52]	292	59%	PSQI
Yennurajalingam USA (2017) [21]	180	62%	PSQI
George, USA (2016) [53]	256	64%	PSQI
Akman, Turkey (2015) [25]	314	40%	PSQI
Nishiura, Tokyo (2015) [55]	50	56%	Athens Insomnia Scale
Mercadante, Italy (2015) [54]	820	61%	Athens Insomnia Scale
Davis, USA (2014) [56]	715	14%	Insomnia Severity Index
Yennurajalingam, USA (2013) [57]	442	75%	Sleep item on a 10-point scale
Delgado-Guay, USA (2011) [58]	101	85%	PSQI

^1^ Patient-reported poor sleep prevalence rate in per cent, PSQI = Pittsburgh Sleep Quality Index.

## Data Availability

Not applicable.
